# Serum carotenoids and Pediatric Metabolic Index predict insulin sensitivity in Mexican American children

**DOI:** 10.1038/s41598-020-79387-8

**Published:** 2021-01-13

**Authors:** Srinivas Mummidi, Vidya S. Farook, Lavanya Reddivari, Joselin Hernandez-Ruiz, Alvaro Diaz-Badillo, Sharon P. Fowler, Roy G. Resendez, Feroz Akhtar, Donna M. Lehman, Christopher P. Jenkinson, Rector Arya, Jane L. Lynch, Jose A. Canas, Ralph A. DeFronzo, Daniel E. Hale, John Blangero, Juan Carlos Lopez-Alvarenga, Ravindranath Duggirala, Jairam K. P. Vanamala

**Affiliations:** 1 South Texas Diabetes and Obesity Institute, Department of Human Genetics, School of Medicine, University of Texas Rio Grande Valley, Edinburg, TX USA; 2 Department of Food Science, Purdue University, West Lafayette, IN USA; 3 Clinical Pharmacology Unit, Hospital General de México Dr. Eduardo Liceaga, Mexico City, Mexico; 4 School of Public Health, University of Texas Health Houston, Houston, TX USA; 5 Department of Medicine, School of Medicine, University of Texas Health San Antonio, San Antonio, TX USA; 6 Department of Pediatrics, School of Medicine, University of Texas Health San Antonio, San Antonio, TX USA; 7 Johns Hopkins All Children’s Hospital, St. Petersburg, FL 33701 USA; 8 Department of Pediatrics, Penn State Health Milton S. Hershey Medical Center, Hershey, PA USA; 9 Department of Food Science, Pennsylvania State University, University Park, PA USA; 10 Department of Plant Science, Pennsylvania State University, University Park, PA USA

**Keywords:** Type 2 diabetes, Obesity, Genetics research, Risk factors

## Abstract

High concentrations of carotenoids are protective against cardiometabolic risk traits (CMTs) in adults and children. We recently showed in non-diabetic Mexican American (MA) children that serum α-carotene and β-carotene are inversely correlated with obesity measures and triglycerides and positively with HDL cholesterol and that they were under strong genetic influences. Additionally, we previously described a Pediatric Metabolic Index (PMI) that helps in the identification of children who are at risk for cardiometabolic diseases. Here, we quantified serum lycopene and β-cryptoxanthin concentrations in approximately 580 children from MA families using an ultraperformance liquid chromatography-photodiode array and determined their heritabilities and correlations with CMTs. Using response surface methodology (RSM), we determined two-way interactions of carotenoids and PMI on Matsuda insulin sensitivity index (ISI). The concentrations of lycopene and β-cryptoxanthin were highly heritable [h^2^ = 0.98, *P* = 7 × 10^–18^ and h^2^ = 0.58, *P* = 1 × 10^–7^]. We found significant (*P* ≤ 0.05) negative phenotypic correlations between β-cryptoxanthin and five CMTs: body mass index (− 0.22), waist circumference (− 0.25), triglycerides (− 0.18), fat mass (− 0.23), fasting glucose (− 0.09), and positive correlations with HDL cholesterol (0.29). In contrast, lycopene only showed a significant negative correlation with fasting glucose (− 0.08) and a positive correlation with HDL cholesterol (0.18). Importantly, we found that common genetic influences significantly contributed to the observed phenotypic correlations. RSM showed that increased serum concentrations of α- and β-carotenoids rather than that of β-cryptoxanthin or lycopene had maximal effects on ISI. In summary, our findings suggest that the serum carotenoids are under strong additive genetic influences and may have differential effects on susceptibility to CMTs in children.

## Introduction

Obesity or overweight affects one in three children in the United States^1^ and is associated with other comorbid conditions such as insulin resistance, hypertension, non-alcoholic fatty liver disease, obstructive sleep apnea, dyslipidemia, and psycho-behavioral problems^2–9^. A major concern is that about 70% of these children are at risk of developing adult obesity that predisposes them to develop several chronic diseases, including metabolic syndrome (MS)^10,11^. Growing evidence suggests that the accumulation of adipose tissue resulting from a positive energy balance leads to a low-grade pro-inflammatory state and oxidative stress^12,13^. There are marked disparities in adult and childhood obesity between various ethnic groups with increased prevalence in Hispanics and African Americans^14^, which are most likely due to environmental and genetic factors and their interactions^15,16^.

Fruits and vegetables contain phytonutrients such as carotenoids, which are considered anti-obesogenic due to their anti-inflammatory and anti-oxidant properties^17,18^. Carotenoid functionality is most likely mediated through modulation of inflammation-related gene transcription and signal transduction pathways and scavenging reactive oxygen species^17,19–23^. While the U.S. Department of Health and Human Services recommends daily fruit and vegetable consumption^24^, ~ 60% of children aged 1 to 18 years do not meet the suggested levels of intake of 1–2 cups of fruits and 1–3 cups of vegetables^25^ and often low socioeconomic status compromises the quality of their diet^26^. Although Hispanic children and adolescents may have higher fruit and vegetable intake compared with African–American and white youth^27^, their relative serum carotenoid concentrations could be variable^28^, suggesting that additional factors should be taken into consideration for understanding metabolic dysfunction in this high-risk population.

Six carotenoids (α-carotene, β-carotene, β-cryptoxanthin, lycopene, lutein, and zeaxanthin) constitute > 95% of the circulating carotenoids in the human body^29,30^. Chemically, carotenoids are lipophilic polyisoprenoid compounds that are either hydrocarbons (α-carotene, β-carotene, lycopene) or the oxygenated xanthophylls (β-cryptoxanthin, lutein, zeaxanthin)^30^. Among these, α-carotene, β-carotene and β-cryptoxanthin, are considered to be provitamin A carotenoids. As their name implies, they can be further metabolized to retinol and related compounds that function as Vitamin A, which is involved in growth, development, and visual function^31^. Lycopene, lutein, and zeaxanthin are non-vitamin A carotenoids, of which the latter two are intimately involved in blue light filtering in the macular region of the retina^32^. Lycopene is the most efficient singlet oxygen quencher among the carotenoids mentioned above^33^.

Humans cannot synthesize carotenoids and rely on the dietary intake of fruits and vegetables for their supply. There is substantial inter-individual variability in serum and tissue carotenoid levels. Several factors, including age, gender, body weight, physical activity, alcohol consumption, drug use, smoking status, and infectious diseases, may determine this inter-individual variability^34^. Also, several candidate-association and genome-wide association studies have identified genetic variants that could potentially influence carotenoid levels and bioavailability by altering their absorption, cleavage, transport, metabolism, and tissue incorporation^35–38^. The genetic basis for inter-individual variation in carotenoids in Mexican Americans is poorly understood. Our recent data showed that serum levels of α-carotene and β-carotene are under strong genetic additive influences in Mexican American children who were at high risk for overweight (53%), obesity (34%), prediabetes (13%), and metabolic syndrome (19%)^39,40^ and that they have phenotypic correlations with several cardiometabolic risk traits (CMTs)^39^. Our recent studies also demonstrated that genetic factors might influence positive phenotypic correlations between β-carotene and HDL cholesterol and negative phenotypic correlation between β-carotene and waist circumference and body mass index^39^. In the current study, our primary objective was to examine the genetic basis of lycopene and β-cryptoxanthin variability among these children and their correlation with CMTs. Importantly, we assessed the interactions between the four carotenoids and a Pediatric Metabolic Index (PMI) which, incorporates adiposity and lipid measures in predicting insulin sensitivity using the response surface methodology.

## Materials and methods

### Subjects

The current study is a family-based cross-sectional study in Mexican American children to determine the heritability of two different carotenoids, β-cryptoxanthin and lycopene, and their association with CMTs. The participants of this study were Mexican American children and adolescents (N = 673, age 6–17 years old), who were recruited as part of the San Antonio Family Assessment of Metabolic Risk Indicators (SAFARI) study^40^. These children were from 401 MA nuclear families/sibships, which were embedded within extended families that we recruited previously as part of three well established genetic epidemiological studies in San Antonio, TX, and vicinity^40^. Many of the children belonged to predominantly low-income extended families. Each nuclear family/sibship had ~ 2 children (range 1–5 children), and the children from all the families generated a total of 3664 relative pairs. As described previously, this study aimed to assess CMTs and their genetic and environmental bases in MA children in San Antonio, Texas. The present study excluded three children who had type 2 diabetes; thus, it involved 670 non-diabetic children. The Institutional Review Board of the University of Texas Health, San Antonio, Texas, reviewed and approved all the research protocols. For each child, written informed consent was obtained from one or both parents, and for children seven years or older, a signed assent was obtained. All studies were performed in accordance with the relevant guidelines and regulations.

### Phenotype data

Extensive information such as family history, socio-demographic characteristics, and environmental data were collected using questionnaires at the clinic or home interviews as described in detail previously^40^. All physical, clinical, and laboratory assessments were performed at the Children’s Center of the Texas Diabetes Institute (TDI), and some laboratory assessments using serum samples were carried out at the Texas Biomedical Research Institute, San Antonio, TX, the USA. as previously reported^40^. Aliquots of biospecimens (e.g., fasting serum samples) collected at the same clinic visits of the children were prepared and promptly frozen at − 70 ºC for future use. The following ten obesity-related quantitative traits were used for estimating the phenotypic, genetic, and environmental correlations with serum carotenoid levels: waist circumference, body mass index (BMI), fat mass assessed by dual X-ray absorptiometry (DXA), blood pressure [systolic (SBP) and diastolic blood pressure (DBP)], fasting plasma glucose, fasting serum specific insulin, the homeostasis model of assessment-insulin resistance (HOMA-IR), HDL cholesterol (HDL-C), and triglycerides. As described previously, serum carotenoids were measured with the available serum samples using Waters’ ultra-performance liquid chromatography-photodiode array (UPLC-PDA)^39^. The carotenoid standards were obtained from Sigma-Aldrich (Saint Louis, MO, USA). The α- and β-carotenoid data were previously reported^39^, and measurements of lycopene and β-cryptoxanthin were obtained for this study (N =  ~ 580). Both dietary intake of carotenoid levels as assessed by the self-reported Block Kids Food Frequency Questionnaire (FFQ) and serum carotenoid data were available for a subset of our SAFARI children (N =  ~ 440). The obtained FFQ information was based on 78 questions regarding the consumption of food/beverage items (frequency and amounts consumed) in the previous week^41,42^. The Block FFQs were analyzed by NutritionQuest (Berkeley, CA). Adjustments were made to carotenoid concentrations using age, sex, *ln* BMI, total energy intake (kcals), and total cholesterol as covariates to assess correlations between dietary intake of carotenoid and serum carotenoid levels^43,44^.

### Statistical analyses

#### Variance components analysis

The genetic basis of lycopene and β-cryptoxanthin were determined using a variance-components approach (VCA) as implemented in the computer program SOLAR^45^. Briefly, in a simple model, variances or covariances between relatives as a function of the genetic relationships can be specified, and the proportion of phenotypic variance that is attributed to (additive) genetic influences (i.e., heritability, h^2^) is estimated from the components of variance. A likelihood ratio test was used to test whether the heritability of a given carotenoid was significant (P ≤ 0.05). The lycopene and β-cryptoxanthin values were inverse normalized for the genetic analyses, and all analyses accounted for covariate effects (e.g., age and sex). We reanalyzed the data to determine the heritability estimate of a given carotenoid after accounting for the dietary intake of the same carotenoid as an additional covariate using a sub-set of our data.

#### Bivariate genetic analysis

Bivariate genetic analysis was used to determine the phenotypic, genetic, and environmental correlations between the carotenoids and the ten CMTs. In this approach, a given phenotypic correlation (ρ_P_) between a pair of phenotypes (e.g., lycopene and BMI) is partitioned into additive genetic (ρ_G_) and environmental (ρ_E_) correlations. The phenotypic correlation (ρ_P_) between a pair of traits is given by:

$$ \rho_{P} = \sqrt {h_{1}^{2} h_{2}^{2} \rho_{G} } + \sqrt {e_{1}^{2} e_{2}^{2} \rho_{E} } $$

where ρ_P_ = the phenotypic correlation; ρ_G_ = the additive genetic correlation; ρ_E_ = the random environmental correlation; $h_{1}^{2}$ = the heritability of trait 1; $h_{2}^{2}$ = the heritability of trait 2; $e_{1}^{2}$ = equal to 1 − $h_{1}^{2}$; $e_{2}^{2}$ = equal to 1 − $h_{2}^{2}$. The significance (P ≤ 0.05) of the phenotypic, additive genetic, and random environmental correlation was determined using likelihood ratio tests. The additive genetic correlation (ρ_G_) is a measure of the shared genetic basis of the two traits (i.e., pleiotropy).

#### Pediatric Metabolic Index (PMI)

PMI was based on two different adiposity-related components obtained from 396 Mexican children^46^. It was shown to be correlated well with HOMA-IR, Matsuda ISI, and hepatic enzymes and could be used to identify children who are at increased risk for cardiometabolic diseases. The first component of PMI was based on the distribution of adipose tissue, and the second component was based on adipose dysfunction. For the second component, median values from the normal-weight children in the population served as the cut-offs with considerations for age (10 years) and sex. PMI index for SAFARI data was calculated as follows:

♀ < 10 years:

$$\left(BMI \;\; z \;\; score\right)+ \left\lfloor\left(\frac{WC}{36.13+(2.3*Age)}\right)\times \left(\frac{TG}{0.88}\right)\times \left(\frac{1.32}{HDL-C}\right)\right\rfloor$$

♀ > 10 years:

$$\left(BMI \;\; z \;\; score\right)+ \left\lfloor \left(\frac{WC}{36.13+(2.3*Age)}\right)\times \left(\frac{TG}{1.04}\right)\times \left(\frac{1.34}{HDL-C}\right) \right\rfloor$$

♂ < 10 years:

$$\left(BMI \;\; z \;\; score\right)+ \left\lfloor \left(\frac{WC}{44.08+(1.48*Age)}\right)\times \left(\frac{TG}{0.77}\right)\times \left(\frac{1.38}{HDL-C}\right)\right\rfloor$$

♂ > 10 years:

$$\left(BMI \;\; z \;\; score\right)+ \left\lfloor \left(\frac{WC}{44.08+(1.48*Age)}\right)\times \left(\frac{TG}{1.06}\right)\times \left(\frac{1.30}{HDL-C}\right)\right\rfloor$$

The abbreviations in the above equations are defined as follows: TG = fasting triglycerides (mmol/L); HDL-C: HDL cholesterol (mmol/L); WC = waist circumference (cm); and BMI = Body Mass Index expressed as Z-score). Age is in years.

#### Response surface methodology (RSM)

The response surface analysis with quadratic effects was used to determine two-way interactions of serum carotenoids and PMI on Matsuda insulin sensitivity index (ISI), a better measure of IR compared to HOMA-IR^40^. Statistical analysis was performed using STATISTICA (version 7).

## Results

### Serum carotenoid levels are highly heritable

A total of 670 non-diabetic children were included in the study after excluding three children with T2D. The serum levels of lycopene and β-cryptoxanthin were highly heritable (lycopene, h^2^ = 0.98, P = 7.3 × 10^–18^; β-cryptoxanthin, h^2^ = 0.58, P = 1 × 10^–7^). We repeated the heritability analysis of a given trait after adjusting for dietary intake of the same carotenoid using a subset of our data based on the availability of the dietary information. Prior to the reanalysis of the data, we found low but significant correlations between dietary intake of carotenoids and serum carotenoid levels as follows: β-cryptoxanthin (ρ ± S.E., P-value): 0.14 ± 0.05, 0.003 and lycopene: 0.17 ± 0.05, 0.0004. However, our reanalyzed data revealed minimal changes in our heritability estimates, in turn suggesting that the original heritability estimates of β-cryptoxanthin and lycopene are largely related to post-absorption-derived concentrations of carotenoids. We previously published the heritabilities of the CMTs as well as the serum α-carotene and β-carotene levels from SAFARI children^39,40^ and are included in Table 1 to aid in data description and additional analyses and interpretation.

**Table 1:** Characteristics of the 670 non-diabetic SAFARI children and heritability estimates for selected cardiometabolic risk traits and carotenoids.

Variable	N	Mean ± SD or%	h^2^ ± SE	*P*-value	References
Girls	670	49.3	–	–	–
Age (years)	670	11.5 ± 3.5	–	–	–
Overweight	670	52.7	–	–	–
Obese	670	33.6	–	–	–
Pre-diabetes	630	13.2	–	–	–
MS^a^	625	18.7	–	–	–
Acanthosis nigricans	661	33.1	–	–	–
BMI (kg/m^2^)	670	22.7 ± 6.5	0.75 ± 0.11	1.1 × 10^–11^	Fowler et al.^40^
Waist circumference (mm)	664	764.5 ± 179.7	0.63 ± 0.12	3.0 × 10^–8^	Fowler et al.^40^
Fat mass (kg) [DXA]	634	16.0 ± 11.1	0.69 ± 0.12	1.8 × 10^–9^	Fowler et al.^40^
Fasting glucose (mg/dl)	630	89.5 ± 7.5	0.39 ± 0.11	6.3 × 10^–5^	Fowler et al.^40^
Fasting insulin (µIU/ml)	626	13.6 ± 9.4	0.55 ± 0.11	2.0 × 10^–7^	Fowler et al.^40^
HOMA-IR	622	2.0 ± 1.3	0.60 ± 0.11	1.8 × 10^–8^	Fowler et al.^40^
HDL cholesterol (mg/dl)	623	45.8 ± 10.9	0.64 ± 0.12	2.9 × 10^–8^	Fowler et al.^40^
Triglycerides (mg/dl)	623	74.9 ± 39.8	0.77 ± 0.11	8.8 × 10^–13^	Fowler et al.^40^
SBP (mmHg)	670	104.1 ± 9.7	0.66 ± 0.11	1.0 × 10^–10^	Fowler et al.^40^
DBP (mmHg)	670	63.2 ± 7.0	0.64 ± 0.11	9.7 × 10^–10^	Fowler et al.^40^
α-carotene (µmol/L)^b,c^	565	0.60 ± 0.97	0.81 ± 0.12	6.7 × 10^−11^	Farook et al.^39^
β-carotene (µmol/L)^b,c^	572	0.48 ± 0.59	0.90 ± 0.11	3.6 × 10^−15^	Farook et al.^39^
Lycopene (µmol/L)^b,c^	590	0.41 ± 0.58	0.98 ± 0.10	7 × 10^–18^	This study
β-cryptoxanthin (µmol/L)^b,c^	593	0.51 ± 0.42	0.58 ± 0.12	1 × 10^–7^	This study

BMI, body mass index; DXA, dual X-ray absorptiometry, HOMA-IR. the homeostasis model of assessment-insulin resistance; HDL cholesterol, high-density lipoprotein cholesterol; SBP, systolic blood pressure; DBP, diastolic blood pressure.

^a^MS = metabolic syndrome as defined in Fowler et al.^40^.

^b^Traits were inverse-normalized for the genetic analyses.

^c^Traits were adjusted for the significant covariate effects of age and sex terms for the genetic analyses.

### Serum carotenoid levels show different patterns of correlations with CMTs

**Figure 1:**
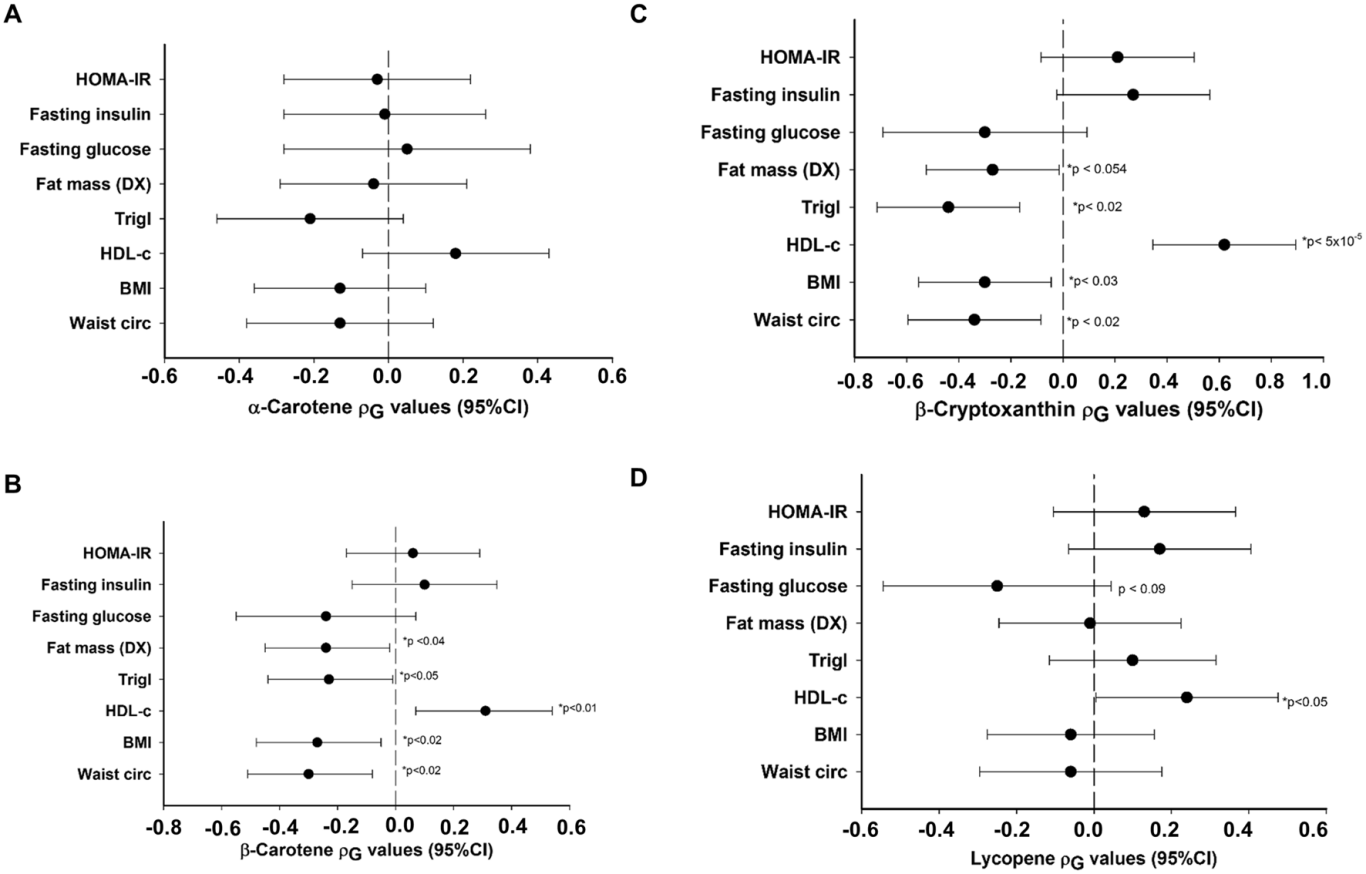
Genetic correlation coefficients (ρG) from α-carotene (**A**), β-carotene (**B**), β-cryptoxanthin (**C**), and lycopene (**D**) and their correlation with CMTs. The solid dots show the estimated rho coefficient; bars correspond to 95% confidence intervals, and the dotted vertical line corresponds to the absence of correlation (ρG = 0). Both carotenoids show a positive correlation with HDL-C. Several obesity-related traits showed a negative correlation with β-carotene and β-cryptoxanthin. If the FDR correction is applied, the significant P-value threshold is < 0.02 (corresponding to a nominal P-value < 0.05). For simplicity, the data for SBP and DBP are not shown.

The results of phenotypic (ρP), genetic (ρG), and environmental (ρE) correlations between lycopene and β-cryptoxanthin and the CMTs are shown in Fig. 1 and Tables 2, 3. For comparative purposes, we also included our previously published data on α-carotene (Fig. 1A) and β-carotene (Fig. 1B). Both β-cryptoxanthin and lycopene showed a significant positive correlation with HDL-C. However, for the other traits, the two carotenoids showed distinct phenotypic and genetic correlations. As shown in Table 2, a significant negative phenotypic correlation (ρ_P_) was found between lycopene and fasting glucose (ρ_P_  = − 0.08; for example, with each S.D. increment of lycopene, the fasting glucose decreased by 5 mg/dL). In contrast, the ρ_P_ between lycopene and HDL-C was positive (ρ_P_ = 0.18; for example, for each S.D. increment of lycopene, HDL-C increased by 2.1 mg/dL) and significant. Likewise, as shown in Table 3, β-cryptoxanthin exhibited a significant positive ρ_P_ of 0.29 with HDL-C. However, it exhibited significant negative ρ_P_s with waist circumference, BMI, triglycerides, and fat mass, which ranged from − 0.25 (waist circumference) to − 0.18 (triglycerides). When the ρ_P_s were partitioned into ρ_G_s and ρ_E_s, only ρ_G_s between β-cryptoxanthin and waist circumference (− 0.34), B.M.I. (− 0.30), triglycerides (− 0.44), and HDL-C (0.62), but not ρ_E_s with the same traits, were statistically significant. In the case of lycopene, only the ρ_G_ with HDL-C was significant. When corrected for multiple testing using the Benjamini–Hochberg procedure^47^, only the phenotypic correlation with HDL-C remained significant for lycopene (i.e., Benjamini–Hochberg procedure-based False Discovery Rate (FDR) adjusted P-value < 0.02 corresponds to a nominal P-value < 0.05). In the case of β-cryptoxanthin, the phenotypic correlations with waist circumference, BMI, HDL-C, triglycerides, fat mass, and systolic blood pressure and genotypic correlations with HDL-C and triglycerides remained significant after FDR correction. For explanation, a positive genetic correlation between two traits indicates that the same genetic factors increase (or decrease) their levels. In contrast, a negative correlation between the two traits indicates that the same genetic factors increase the levels of one trait and decrease the levels of the other trait.

**Table 2:** Phenotypic (ρ_P_), genetic (ρ_G_) and environmental (ρ_E_) correlations between lycopene and the cardiometabolic risk traits.

Trait pair^a^	ρ_P_ (95% CI)	*P*-value	ρ_G_ (95% CI)	*P*-value	ρ_E_ (95% CI)	*P*-value
Waist circumference	− 0.05 (− 0.128, 0.028)	0.24	− 0.06 (− 0.295, 0.175)	0.61	− 0.02 (− 1,1)	0.97
Body Mass Index^b^	− 0.04 (− 0.138, 0.058)	0.35	− 0.06 (− 0.276, 0.156)	0.61	0.06 (− 1,1)	0.93
HDL cholesterol	0.18 (0.102, 0.258)	**5.69** × **10**^–5^*	0.24 (0.005, 0.475)	**0.05**	− 0.08 (− 1, 0.998)	0.87
Triglycerides^b^	− 0.03 (− 0.128, 0.068)	0.51	− 0.1 (− 0.316, 0.116)	0.39	0.47 (− 1,1)	0.46
Fat mass^b^	− 0.03 (− 0.128, 0.068)	0.52	− 0.01 (− 0.245, 0.225)	0.91	− 0.14 (− 1,1)	0.82
Systolic blood pressure	− 0.03 (− 0.108, 0.048)	0.53	− 0.04 (− 0.275, 0.195)	0.70	0.066 (− 0.9728,1)	0.90
Diastolic blood pressure	− 0.03 (− 0.108, 0.048)	0.54	− 0.02 (− 0.255, 0.215)	0.89	− 0.03 (− 1,1)	0.58
Fasting glucose^b^	− 0.08 (− 0.158, − 0.002)	**0.04**	− 0.25 (− 0.544, 0.044)	0.09	0.33 (− 0.67,1)	0.38
Fasting insulin^b^	0.01 (− 0.068, 0.088)	0.78	0.17 (− 0.065, 0.405)	0.18	− 0.72 (− 1, 0.828)	0.12
HOMA-IR^c^	0.02 (− 0.058, 0.098)	0.70	0.13 (− 0.105, 0.365)	0.27	− 0.59 (− 1, 0.821)	0.24

*HDL* cholesterol, high-density lipoprotein cholesterol, *HOMA-IR* the homeostasis model of assessment-insulin resistance.

Asterisks indicate the p-values that remain significant after applying FDR correction.

Bold values denote statistical significance at *P*-value ≤ 0.05.

^a^All traits were adjusted for the covariate effects of age and sex terms.

^b^Data were log-transformed.

^c^Trait was transformed using inverse normal transformation.

**Table 3:** Phenotypic (ρ_P_), genetic (ρ_G_) and environmental (ρ_E_) correlations between β-cryptoxanthin and the cardiometabolic risk traits.

Trait pair^a^	ρ_P_ (95% CI)	*P*-value	ρ_G_ (95% CI)	*P*-value	ρ_E_ (95% CI)	*P*-value
Waist circumference	− 0.25 (− 0.328, − 0.172)	**2.93** × **10**^–9^*	− 0.34 (− 0.595, − 0.085)	**0.02**	− 0.1 (− 0.551, 0.351)	0.67
Body Mass Index^b^	− 0.22 (− 0.2984, − 0.142)	**2.08** × **10**^–7^*	− 0.3 (− 0.555, − 0.045)	**0.03**	− 0.06 (− 0.589, 0.469)	0.82
HDL cholesterol	0.29 (0.212, 0.368)	**1.32** × **10**^–12^*	0.62 (0.346, 0.894)	**5.75** × **10**^–5^*	− 0.19 (− 0.641, 0.261)	0.35
Triglycerides^b^	− 0.18 (− 0.258, − 0.102)	**5.30** × **10**^–5^*	− 0.44 (− 0.714, − 0.166)	0.002*	0.36 (− 0.189, 0.909)	0.14
Fat mass^b^	− 0.23 (− 0.308, − 0.152)	**1.7** × **10**^–7^*	− 0.27 (− 0.525, − 0.015)	0.054	− 0.16 (− 0.611, 0.291)	0.51
Systolic blood pressure	− 0.07 (− 0.148, 0.008)	0.01*	− 0.13 (− 0.424, 0.164)	0.35	0.03 (− 0.362, 0.422)	0.87
Diastolic blood pressure	− 0.07 (− 0.148, 0.008)	0.10	− 0.18 (− 0.474, 0.114)	0.23	0.1 (− 0.292, 0.492)	0.60
Fasting glucose^b^	− 0.09 (− 0.168, − 0.0116)	**0.04**	− 0.3 (− 0.692, 0.092)	0.12	0.09 (− 0.204, 0.384)	0.54
Fasting insulin^b^	− 0.07 (− 0.148, 0.008)	0.10	0.27 (− 0.024, 0.564)	0.15	− 0.56 (− 0.932, − 0.188)	0.002
HOMA-IR^c^	− 0.07 (− 0.148, 0.008)	0.11	0.21 (− 0.084, 0.504)	0.16	− 0.52 (− 0.932, − 0.108)	0.009

*HDL* cholesterol, high-density lipoprotein cholesterol, *HOMA-IR* the homeostasis model of assessment-insulin resistance.

Asterisks indicate the p-values that remain significant after applying FDR correction.

Bold values denote statistical significance at *P*-value ≤ 0.05.

^a^All traits were adjusted for the covariate effects of age and sex terms.

^b^Data were log-transformed.

^c^Trait was transformed using inverse normal transformation.

### Differential interactions of individual serum carotenoid levels with insulin sensitivity

**Figure 2:**
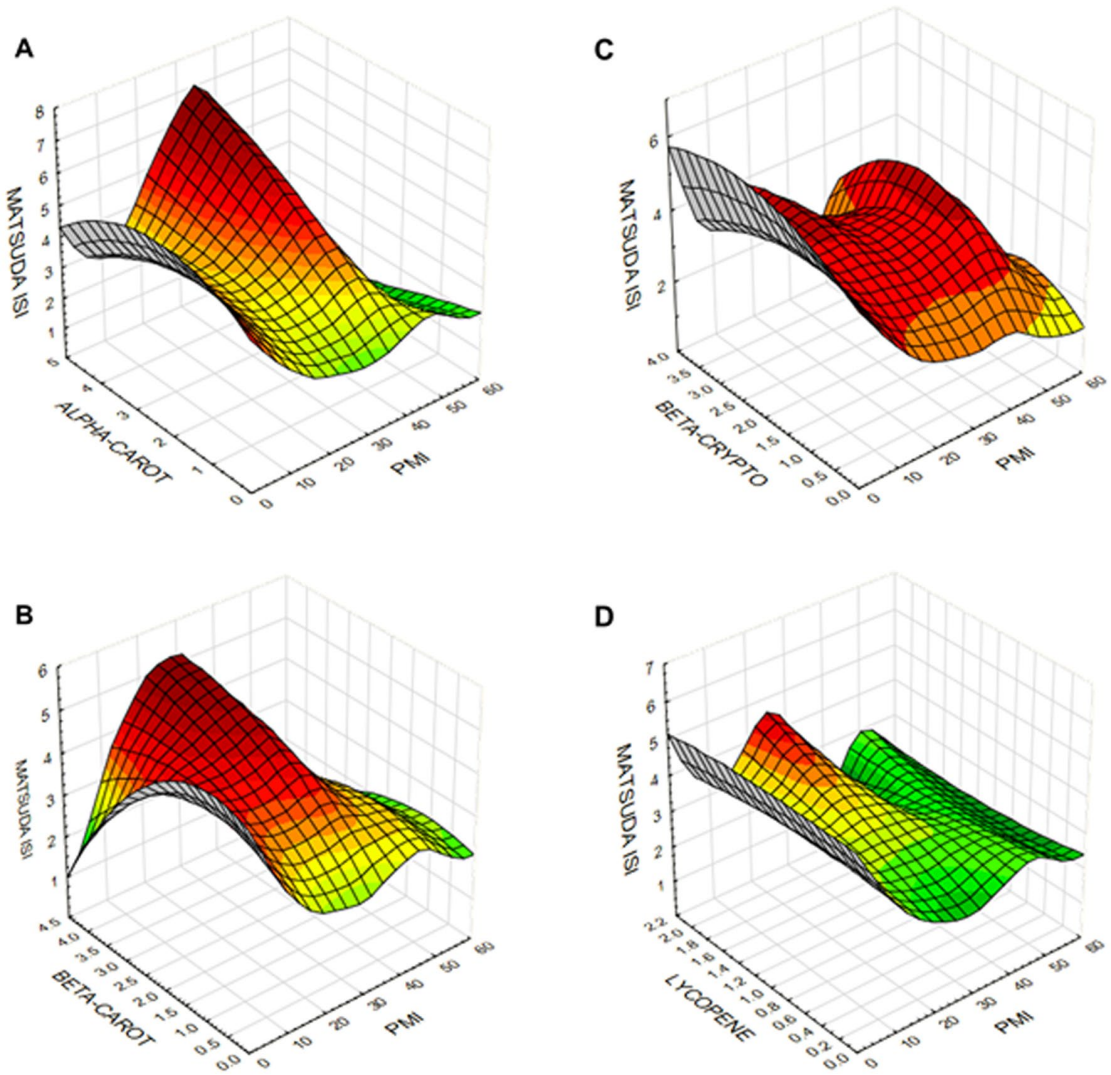
Response surface contour plots showing the relationship of α-carotene (**A**), β-carotene (**B**), β-cryptoxanthin (**C**), and lycopene (**D**) and PMI with Matsuda ISI. The contour plots are colored to aid in better visualization of the graphics.

We used RSM to determine two-way interactions of serum carotenoids and PMI on Matsuda ISI. RSM generates a surface fitted three-dimensional plot using a distance-weighted least-squares procedure while minimizing the variance of estimators by a polynomial regression model weighted for the inverse of the variances. The plots shown in Fig. 2 represent the interactions between serum carotenoid concentrations with PMI while showing the extent of insulin sensitivity measured by Matsuda ISI. Interestingly, the resulting function is nonlinear, and a maximum response was obtained with increasing serum α- and β- carotenoids (Fig. 2A,B), but not with β-cryptoxanthin or lycopene (Fig. 2C,D). However, there is a significant decrease in Matsuda ISI with lower levels of α- and β-carotenoids. PMI values between 2 and 4 showed the maximum variability associated with carotenoids.

## Discussion

Our family-based study demonstrates that genetic factors play a crucial role in determining the serum carotenoid concentrations in MA children. Similarly, several other studies reported a range of heritabilities for carotenoids^39,48–50^, given that the heritability is a population-specific parameter. For example, high heritability (r^2^ = 0.98) has been reported for relative peak macular pigment density in monozygotic twins^51^ and moderate heritability (h^2^ = 30.5%) for serum retinol levels in a French family study^49^. A recent study in Older Order Amish adults estimated the heritability of serum lycopene to be 0.38 ± 0.12^48^. While it can be argued that shared environmental influences could have resulted in high heritabilities observed in our study, we believe that these estimates are plausible as most of the children are distributed across large families. These findings are similar to those we found for the α-carotene and β-carotene in the same group of children^39^. The serum levels of β-cryptoxanthin and lycopene consistently showed a positive correlation with HDL-C levels while there was a marked difference in their effects on other CMTs. We previously observed a similar correlation between serum α-carotene and β-carotene and HDL-C^39^. These results are in agreement with the concept that plasma lipoproteins as the primary transporters of carotenoids^52^.

Given that diet is the primary source of serum carotenoids, based on our available data, we assessed the extent to which the observed high heritabilities are related to the dietary intake of carotenoids in our study. Despite the limitations of the FFQ derived estimates of dietary markers, including carotenoids, we found low but highly significant correlations between dietary and serum carotenoids after making appropriate covariate adjustments. Here, it is worth noting that there is no consensus on the nature of the relationship between dietary intake and blood (either serum or plasma) carotenoid concentrations^43,44,53–55^. Also, such relationships may differ by individual carotenoids^43^. Moreover, blood concentrations of carotenoids may be able to better predict disease risk when compared to dietary intake^56^. However, our findings of minimal changes in heritability estimates of carotenoids after adjustments for dietary carotenoids suggest that the observed high heritabilities are mainly related to post-absorption-derived concentrations of carotenoids. This conclusion may not be that surprising since many other factors, aside from diet, related to carotenoid uptake, distribution, metabolism, and excretion, contribute to their levels in the body^34^. Also, it should be noted that carotenoids have long and variable half-lives and follow first-order depletion kinetics^57^.

In this study, β-cryptoxanthin showed a negative correlation with several CMTs, including waist circumference, BMI, triglycerides, and fat mass, but lycopene failed to show any correlation with any of the examined CMTs. Despite strong evidence for the possible role of lycopene in the suppression of adipogenesis, there are conflicting reports on the correlation between lycopene and BMI and related traits^28,58^. Notwithstanding the observation that BMI could influence the association between metabolic syndrome and serum lycopene levels, the associations between lycopene and the metabolic syndrome were only significant for normal-weight and participants who are overweight and not for individuals with obesity in the adult National Health and Nutrition Examination Survey (NHANES) data^59^. A previous study reported higher serum concentrations of α-tocopherol, α-carotene, and trans-β-carotene in Mexican American children were associated with reduced childhood overweight and obesity^60^. While similar associations were found in the SAFARI children with α-carotene and β-carotene^39^, our current study further extends these observations to include β-cryptoxanthin suggesting an overall beneficial effect of serum levels of these three carotenoids.

In a systemic review of multiple studies conducted on anti-oxidant vitamins, Asplund showed that lower cardiovascular risk is associated with high intake of β-carotene (OR = 0.88; 95%CI 0.77–1.01) or high serum/plasma levels of β-carotene (OR = 0.46; 95%CI 0.37–0.58) whereas such effect is not seen in randomized controlled trials with β-carotene supplements (OR = 1.02; 95%CI 0.96–1.08)^61^. Additional studies also have shown that serum carotenoids have anti-oxidant activity, and low levels of serum carotenoids and other anti-oxidants are associated with insulin resistance, T2D, and metabolic syndrome^62–70^. Kinetic analysis of plasma carotenoid concentrations shows extreme variability in absorption curves and plasma clearance rates^71^. While the mechanism of intracellular translocation of carotenoids is not completely understood, recent studies have shown that in addition to passive diffusion, transporters such as SR-BI may play a role in their absorption^72^. Nevertheless, the overall carotenoid bioaccessibility is considered to be poor and variable among individuals, and such variability may contribute to the carotenoid “low responder” and “high responder” phenotypes^72^. Additional factors such as dietary fiber content and composition of gut microbiota may also alter carotenoid absorption, bio-activation, and circulating plasma concentrations. How consumption of carotenoids can regulate gut immune responses and microbiota is a subject of active ongoing research^73^.

We initially developed PMI as an omnibus clinical measure that included adiposity, HDL-C, and triglyceride concentration to assess the relative metabolic health of children in a population after adjusting for age and sex. Remarkably, we found that serum α- and β- carotenoid concentrations were inversely associated with PMI in the SAFARI children using the RSM approach. However, their protective effects were modified by underlying metabolic conditions and were diminished in the presence of metabolic impairment (PMI > 4). Some previous studies showed both dietary and plasma β-carotene, but not lycopene and β-cryptoxanthin, may positively associate with insulin sensitivity. Sluijs et al. reported that diets high in β-carotene were protective against T2D [Hazard Ratio quartile 4 versus quartile 1 (HRQ4): 0.78 (95%CI 0.64, 0.95), P-linear trend 0.01]^67^. In contrast, they found that the effect of serum α-carotene on T2D was marginal [HRQ4 of 0.85 (95%CI 0.70,1.03), and P-linear trend 0.05], whereas other carotenoids did not show any association. In a study conducted on non-diabetic adult subjects with obesity, plasma β-carotene concentrations were positively associated with insulin sensitivity, as assessed by HOMA-IR, whereas such associations were not detected with lycopene and lutein/zeaxanthin^58^. These studies, along with our present study, highlight the fact that various carotenoids may have non-overlapping and differential associations with insulin sensitivity in children, as previously reported in adults. However, the protective associations of the α- and β-carotenoids were not detectable in the presence of metabolic dysfunction. This concept needs further exploration, given our previous finding that fruit and vegetable juice concentrate supplementation could lead to increased serum β-carotene levels and improved insulin resistance in overweight prepubertal boys^74^.

A balanced diet should incorporate a variety of fruits and vegetables as sources of different carotenoids. Orange colored vegetables such as carrots and green vegetables such as broccoli and spinach are rich sources of α-carotene and β-carotene^75,76^. Red-colored vegetables such as tomatoes are lycopene-rich, and orange-colored fruits such as mandarins are rich in β-cryptoxanthin^75,76^. Our perplexing finding that the serum levels of β-cryptoxanthin, but not lycopene, being protective against many CMTs in the SAFARI children has prompted us to examine this issue further. Unlike many nutrients, lycopene’s bioavailability is increased by cooking, and processing and tomato-based products like ketchup and sauces are a common dietary source of lycopene^77^. However, limited data exists on the relative health benefits of consuming whole tomatoes versus processed tomato products and lycopene supplements. Thus rigorous, well-controlled studies are needed to understand the differential effects of carotenoids in CMTs.

Reinforcing our results that indicate high heritability of serum carotenoid levels, genome-wide association analyses (GWAS) as well as candidate gene association studies (CGAS) have identified several genetic variants that could potentially contribute to inter-individual variability in carotenoid levels in the body^36^. These genetic variants map to various stages in carotenoid metabolism, including absorption, conversion, fasting, and post-prandial blood concentrations, and tissue levels. Also, gene variants that alter lipid absorption and metabolism can influence circulating carotenoid levels^78^. For example, genetic variants in *BCO1, ABCA1, APOB, and LPL,* have been implicated in fasting blood β-carotene concentrations, whereas genetic variants in *RBP4* in fasting blood retinol concentrations (reviewed by Borel and Desmarchelier^79^). Similarly, serum lycopene and β-cryptoxanthin levels may also be genetically determined^78,80^. Such genetic variation could potentially lead to differences in carotenoid metabolism in global populations. However, irrespective of such genetic variability, consumption of a wide variety of fruits and vegetables need to be encouraged, given the broad protective effects of carotenoids on human health.

## Conclusions

While dietary intake of fruits, vegetables, or supplements is critical for serum carotenoid levels, they are also determined by genetic influences that may alter their absorption, transport, tissue concentration, and utilization of the carotenoids. Other modifying factors may include the differences in gut microbiota, socioeconomic strata, other metabolic conditions, and their interactions. Importantly, our findings suggest a connection between concentrations of certain serum carotenoids and insulin sensitivity patterns; increased serum concentrations of α- and β- carotenoids were shown to have maximal effects on insulin sensitivity. In summary, our study reveals that the serum carotenoids are under strong additive genetic influences; and, may have differential effects on susceptibility to childhood obesity and its related CMTs in children and adolescents.
